# Secondary Plasma Cell Leukemia in a Recurrent Multiple Myeloma: Rare Case Scenario

**DOI:** 10.7759/cureus.8456

**Published:** 2020-06-05

**Authors:** Arati A Inamdar, Abraham Loo, Nagy Mikhail, Patrick Lee

**Affiliations:** 1 Pathology, RWJBarnabas Health, Livingston, USA; 2 Pathology and Laboratory Medicine, Rutgers Robert Wood Johnson Medical School, New Brunswick, USA; 3 Pathology, Rutgers Robert Wood Johnson Medical School, New Brunswick, USA; 4 Hematology Oncology, Monmouth Medical Center, Long Branch, USA

**Keywords:** flow cytometry, multiple myeloma, novel immunomodulatory agents, plasmacytoma, plasma cell leukemia

## Abstract

Plasma cell leukemia (PCL) is an aggressive hematological condition characterized by the presence of plasma cells in the peripheral smear. It presents as de novo or may arise from multiple myeloma (MM), and hence is diagnosed as primary or secondary PCL, respectively. We report a case of 79-year-old patient diagnosed with MM two years prior to the admission to our institution with prior treatment with bortezomib, lenalidomide and dexamethasone (VRD) and daratumumab, pomalidomide and dexamethasone. Morphologic examination and flow cytometry studies performed on the peripheral smear demonstrated 45%-55% small to medium atypical plasma cells showing a kappa restriction and dim CD138 expression on flow cytometry analysis. The patient was started on brentuximab vedotin, etoposide, cytoxan and dexamethasone, which resulted in near complete elimination of the atypical plasma cells from the peripheral smear one week after the completion of two cycles. He received three cycles of brentuximab vedotin with a gradual decrease in serum free light chain. However, he eventually developed lethargy, weakness and seizures. The involvement of the central nervous system (CNS) by MM was confirmed with MRI, flow cytometry and cytology of cerebrospinal fluid. The treatment with whole brain radiation and ibrutinib was initiated. Our case report highlights the rare case of aggressive clinical course of MM leading to the development of plasmacytoma of kidney, secondary PCL and eventually spreading to the CNS.

## Introduction

Plasma cell leukemia (PCL) is defined by the presence of >2 × 10^9^/liter circulating plasma cells (CPCs) in the peripheral blood or by a relative plasmacytosis >20% of blood leukocytes [1]. In rare cases (2%-4%), late or advanced stage multiple myeloma (MM) may undergo clonal transformation and develop into secondary plasma cell leukemia (sPCL) [2]. Recent studies have compared the overall survival (OS) of patients with MM with percentage of CPCs in the peripheral blood. No difference in survival is noted between the patients of sPCL with 5%-19% and those with >20% CPCs. Such comparative studies have advocated for a lower threshold of CPCs to define PCL [3-5]. The morphology and immunophenotype of the clonal plasma cells seen in primary PCL and sPCL are similar; hence,a clinical history of MM is crucial in establishing a diagnosis of sPCL. sPCL has a dismal prognosis with a median OS of only seven months with standard chemotherapy [6]. MM with t (11:14) is seen in 15%-20% of all cases and is considered as an intermediate risk with often unpredictable outcome [7]. We present a unique case of a 79-year old male with a past history of relapse/refractory MM evolving from monoclonal gammopathy of undetermined significance (MGUS) within two years followed by a relapse with plasmacytoma of the kidney. He was admitted to our institution for further management of his aggressive MM and diagnosed with PCL. Despite initial response to the treatment regimen, central nervous system (CNS) involvement by MM was revealed within four months of initial presentation. Our report highlights the rare case of aggressive form of secondary form of PCL with plasmacytoma of kidney and CNS involvement. 

## Case presentation

**Figure 1:**
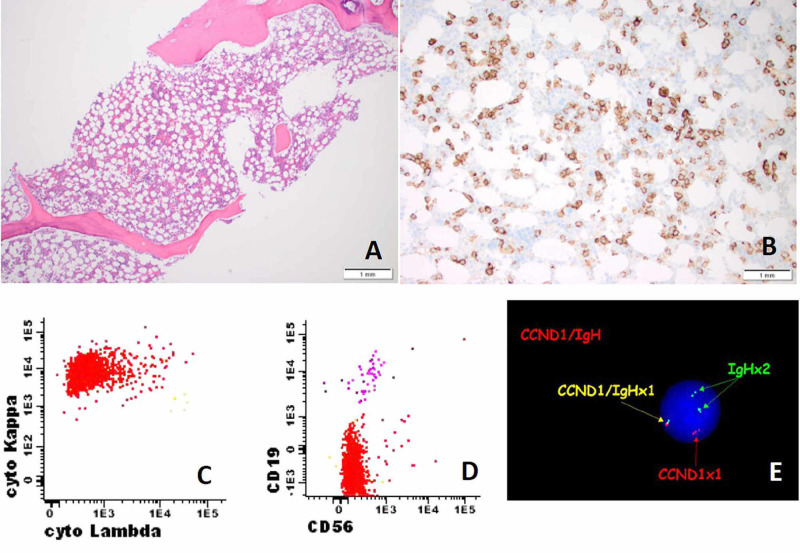
Bone marrow biopsy, flow cytometry (CD38 gating) and FISH study for multiple myeloma. (A) Bone marrow biopsy demonstrated normocellular marrow with 20%-30% cellularity along with increased CD138-positive plasma (brown colored) cells (B). (C) Bone marrow aspirate flow cytometry studies showed kappa-restricted clone, negative for CD56 (D). (E). FISH analysis using break apart probe on bone marrow aspirate detected t (11:14) translocation. FISH, Fluorescence in situ hybridization

Our case report involves a 79-year-old male with a diagnosis of MGUS at outside institution who underwent bone marrow biopsy due to persistent anemia and hypogammaglobulinemia at another institution. The biopsy specimen demonstrated normocellular marrow with 20%-30% cellularity along with decreased myeloid:erythrocyte (M:E) ratio due to a mild erythroid hyperplasia and mild granulocytic hypoplasia (Figure 1A). A CD138 immunohistochemical stain demonstrated a marked increase (>10%) in plasma cells (Figure 1B). Flow cytometry studies demonstrated monoclonal kappa-positive plasma cell population, which were negative for CD56 and comprised 0.9% of total events (Figures 1C, 1D). Fluorescence in situ hybridization (FISH) analysis demonstrated a t(11:14) (Figure 1E) without any other cytogenetic abnormalities such as p53, deletion of 1p (CDKN2C), additional copy of 1q (CKS1B) or deletion of retinoblastoma 1. Laboratory findings showed elevated lactate dehydrogenase (LDH) with low calcium. Based on these findings, the patient was diagnosed with MM and treated with bortezomib, lenalidomide and dexamethasone (VRD). 

**Figure 2:**
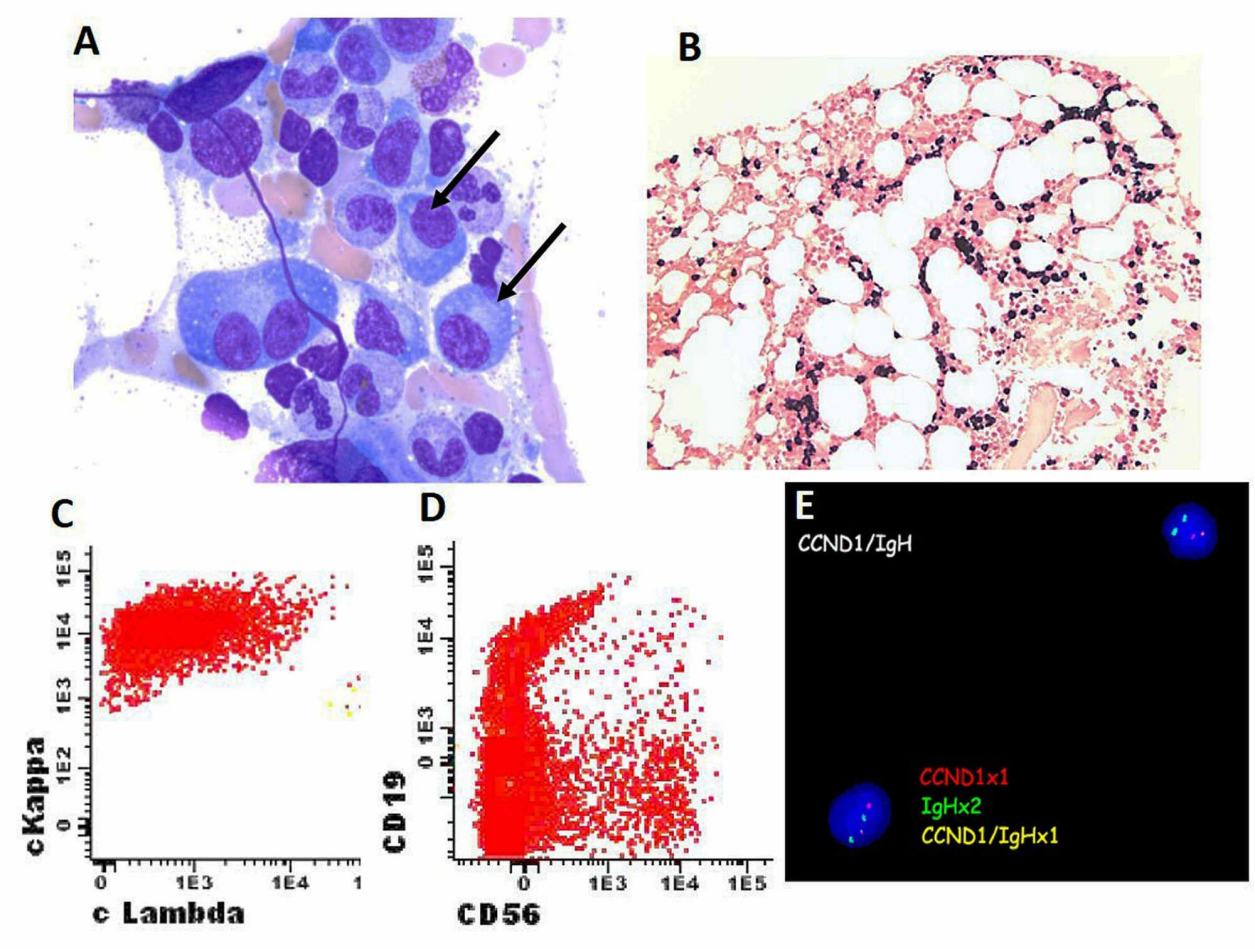
Bone marrow biopsy, flow cytometry and FISH study for recurrent multiple myeloma. (A) Bone marrow biopsy demonstrated plasma cells (arrows) and predominantly kappa expression by plasma (black colored) cells (B). (C). Flow cytometry studies showed a kappa-restricted clone, which was positive for CD56 (D). (E). FISH analysis using break apart probe on bone marrow aspirate detected t (11:14) translocation. FISH, Fluorescence in situ hybridization

He underwent another bone marrow biopsy almost a year later, which revealed persistent/recurrent kappa monoclonal plasma cells involving 20%-30% of marrow cellularity (Figure 2A). The abnormal plasma cells demonstrated weak CD138 staining by immunohistochemical stain. Kappa and lambda in situ hybridization stains demonstrated a marked kappa restriction (Figure 2B). Flow cytometry studies performed on the aspirate upon gating CD38-positive cells demonstrated 12% plasma cells with a kappa restriction and partial CD19 and CD56 expression (Figures 2C, 2D). Cytogenetics/FISH studies again showed t(11:14) abnormality only (Figure 2E).

**Figure 3:**
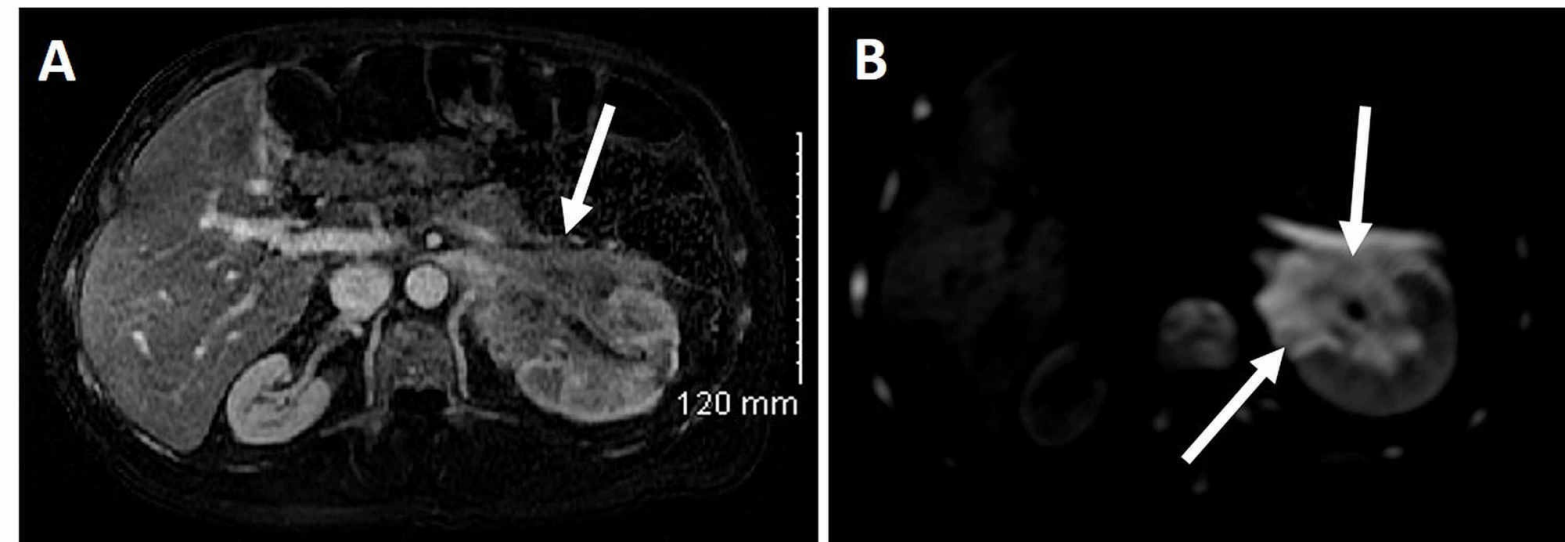
MRI of abdomen and pelvis. (A, B) A large ill-defined mass (arrows) was identified in the left kidney near hilum as visualized via T-1 and T-2 weighted images, respectively.

The patient received six cycles of daratumumab, pomalidomide and dexamethasone. The patient then developed severe abdominal pain. MRI of the abdomen and pelvis revealed a large mass in the left kidney near the hilum measuring 7.0 x 6.0 x 5.0 cm (Figure 3). A biopsy of the lesion showed diffuse sheet of atypical plasmacytoid cells with immunophenotypic profile consistent with plasmacytoma, positive for CD138, CD56, CD30 and kappa-ish, and negative for CD5, PAX5, CD20 and CD117. Flow cytometry studies demonstrated clonal plasma cells with a cytoplasmic kappa light chain (KLC) restriction and positivity for CD138, CD56, CD4 and kappa.

**Figure 4:**
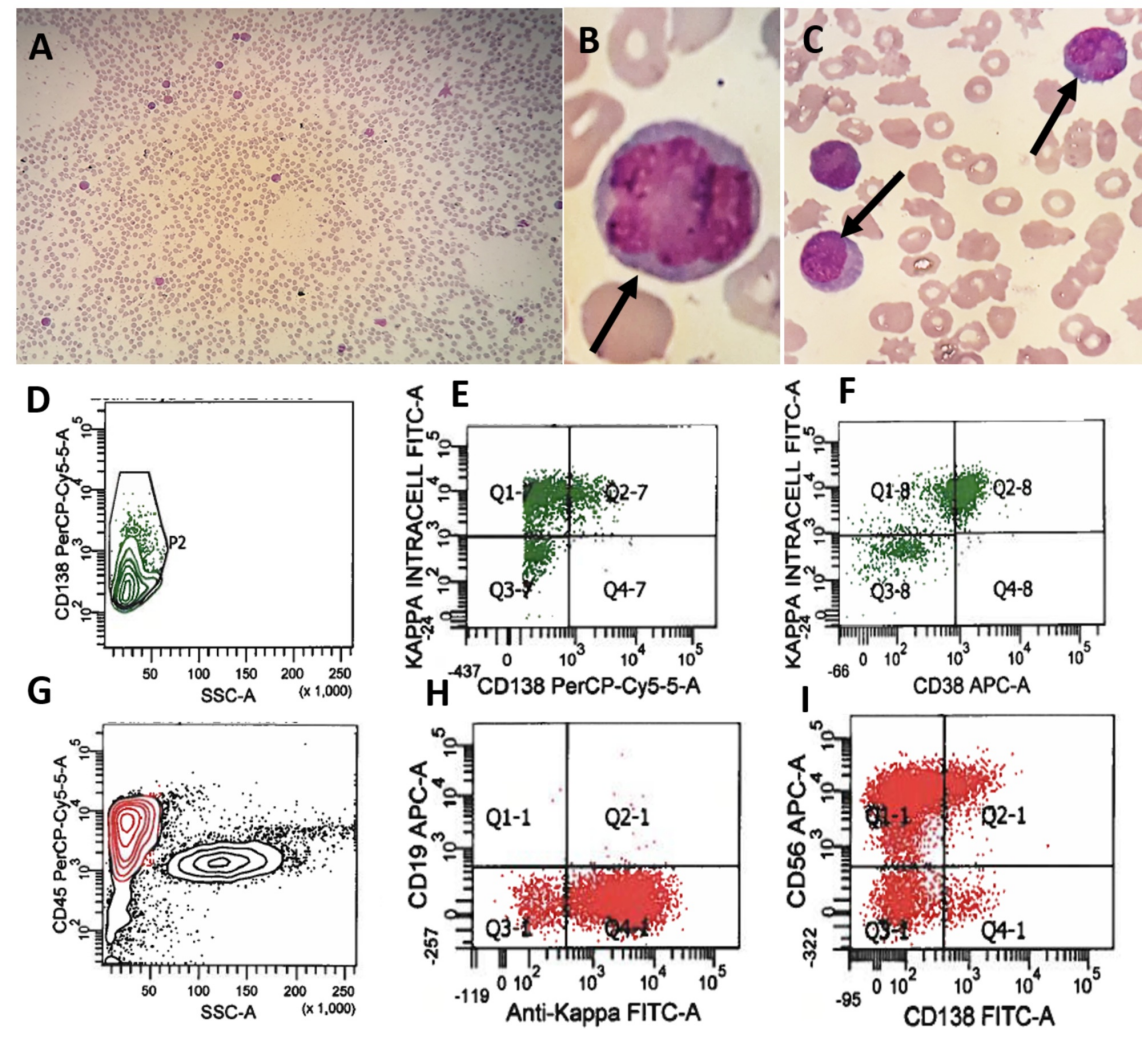
Plasma cell leukemia in recurrent multiple myeloma. (A-C) Peripheral smear analysis demonstrated leukocytosis (A) and abnormal plasma cells (B, C). (D) Flow cytometry studies showed 22.4% CD138 dim-positive cells with kappa restriction (E) and dim-positive expression of CD38 (F). (G) The zebra plot showed abnormal cell population with kappa restriction (H) with expression of CD56 and CD138 (dim) markers (I).

With the prior history of recurrent MM not achieving remisssion and plasmacytoma of the left kidney, the patient came to our institution with progressively increasing leukocytosis, hypogammaglobulinemia and anemia. On admission, laboratory evaluation of peripheral blood showed a hemoglobin of 9.5 g/dL, a white blood cell count of 27.2 K/mm^3^, which was interpreted by the hematology technicians as 12% atypical lymphocytes, and platelet count of 61 K/mm^3^. Pathologist review of the peripheral smear revealed 45%-55% immature small to large multilobated plasma cells with fine chromatin and moderate to high nuclear:cytoplasmic ratio (Figures 4A-4C). Flow cytometry performed on the peripheral blood confirmed PCL with 22.4% CD138/CD38 (dim-positive) plasma cells, which were also expressing intracellular KLC (Figures 4D-4F). Interestingly, 90.5% of these plasma cells demonstrated surface kappa expression without expression of CD19 (Figures 4G, 4H). Furthermore, 70% of plasma cells showed CD56 expression (Figure 4I). Other relevant laboratory parameters include blood urea nitrogen 29 mg/dL, creatinine 2.05 mg/mL and glomerular filtration rate 32 mL/min/1.73 m^2^. Quantitative serum protein analysis revealed low IgG, IgA and IgM of 127, 14 and 5 mg/dL, respectively. The serum kappa/lambda ratio was 4.94 where kappa and lambda free were 7.9 and 1.6 mg/L, respectively. The patient was started on brentuximab vedotin, etoposide, cytoxan and dexamethasone.

**Figure 5:**
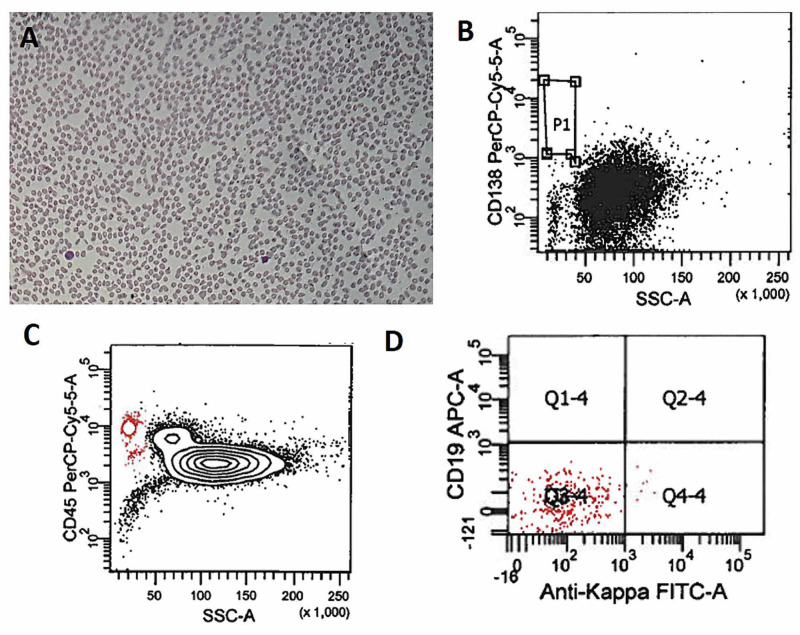
Post therapy effect on plasma cell leukemia. (A) Peripheral smear analysis demonstrated leukocytopenia. (B) Flow cytometry analysis did not show residual CD138-positive cells. (C) The zebra plot did not show residual kappa-positive cells (D).

The peripheral smear examined one week after completion of two cycles of brentuximab vedotin, etoposide, cytoxan and dexamethasone showed leukocytopenia with near complete elimination of plasma cells from the peripheral smear (Figure 5A). Flow cytometry did not detect a residual CD138-positive population or kappa expressing population (Figures 5B-5D). The patient was continued on three cycles of brentuximab vedotin with progressive response with a gradual decrease in serum free light chain (FLC). However, he eventually developed lethargy, left leg weakness and imbalance. The MRI showed multiple metastatic lesions in the brain. He underwent lumbar puncture with cerebrospinal fluid showing atypical cells, but flow cytometry revealed the clonal CD38, CD56, CD45 and intracellular kappa-positive population with aberrant expression of clonal CD4 cells comprising 40% of total events. These findings confirmed the metastatic involvement of patients with MM.

## Discussion

WHO defines MM at least one or more myeloma‐defining events (MDEs) in addition to evidence of either 10% or more clonal plasma cells on bone marrow examination or a biopsy-proven plasmacytoma. MDE consists of established parameters for CRAB features (hypercalcemia, renal failure, anemia or lytic bone lesions) and other biomarkers of malignancy: clonal bone marrow plasma cells of 60% or higher, serum FLC ratio of 100 or higher (provided involved FLC level is ≥100 mg/L) and more than one focal lesion on MRI [2,8].

MGUS is considered as a premalignant condition for MM which devoid of clinical criteria associated with MM. MGUS patients usually have 1% average annual risk of progression to malignant MM. Our patient demonstrated symptom of MM within two years after being diagnosed with MGUS.

PCL is a rare disease and accounts for 1%-2% of all hematological malignancies with a median age of 50-60 years with an approximately equal proportion of male and female patients. Although PCL is defined by the presence of >2 × 10^9^/liter of CPCs in peripheral blood or by a relative plasmacytosis >20% of blood leukocytes, recent studies have advocated the use of a lower threshold of CPCs to define PCL [3-5]. Despite undergoing cycles of combination of bortezomib, lenalidomide, daratumumab, pomalidomide and dexamethasone for MM, our patient failed to achieve remission. Unfortunately, while on treatment regimen, he developed plasmacytoma of kidney (Figure 3). Furthermore, CD30-positive cells were detected in the plasmacytoma via flow cytometry, and hence brentuximab vedotin was added to the regimen. At our institute, during routine peripheral smear review, 45%-55% of abnormal cells with multilobated morphology were seen (Figures 4A-4C). The flow cytometry of peripheral blood confirmed presence of 22.4% CD138 dim-positive and kappa-restricted plasma cells (Figure 4D-4F). These findings were consistent with a diagnosis of sPCL. In our patient, CD38 expression was also dim due to treatment with daratumumab, a anti-CD38 antibody (Figure 4F) [9]. Interestingly, flow cytometry of the peripheral blood also demonstrated dim CD138 expression (Figure 4D). CD138 molecule is known to disintegrate and disappear quickly in the sample, and hence flow cytometry analysis for CD138 is usually underestimated. Furthermore, expression can be lost during the processing of the sample for flow cytometry; hence, the dim CD138 expression may not represent the true biological expression of CD138 in our patient as well [10]. In addition to the cytoplasmic kappa, almost 70% of these abnormal cells also expressed surface kappa of which some were positive for CD56 and CD138 markers (Figure 4G-4I). Usually, plasma cells in PCL and MM patients express cytoplasmic light chain clone but plasma cells have also been shown to express surface as well as cytoplasmic light chain clone in other published reports [10,11]. In addition, multilobated form of plasma cells seen in our patient peripheral smear has also been reported previously [12]. These findings highlight that sPCL may demonstrate abnormal morphology instead of typical plasma cells and require careful assessment of peripheral smear during the management of MM. In addition, the rapid progression from MM to plasmacytoma and then sPCL suggest the aggressive form of MM and require multidisciplinary management and treatment regimen keeping in the mind the phenotype of the abnormal lesion cells. 

sPCL usually developed in association with translocations of immunoglobulin heavy chain (IgH) translocation with other chromosomal partners (4p16, 6p21, 11q13, 16q23 and 20q11) with or without associated mutation/deletion in the chromosomes and other genes such as NRAS, KRAS, p53, MYC, NF-kB, etc [6]. Especially, higher incidence of t(11; 14) (q13; q32) and other chromosomal abnormalities, such as del (17p13), del (1p21), ampl (1p21), t (14; 12) and t(4:14), have also been reported [13]. 

Although transformation of MM to sPCL is highly unpredictable, failure to respond to initial as well as elevated LDH, low serum albumin, elevated beta 2 microglobulin, hypercalcemia, advance age and increase plasma cells at the time of diagnosis of MM are some of the risk factors for progression to sPCL [6]. PCL with t(11;14) has highly unpredictable clinical course [7]. Studies indicate that a reduction in CPCs by 50% within 10 days of treatment and to have no CPCs within four weeks of treatment are associated with relatively longer survival [6,14]. Furthermore, for such patients, addition of bcl-2 inhibitor, venetoclax has recently shown promise [15]. Our patient had a complete workup at the time of diagnosis of initial MM, which demonstrated high LDH, abnormal serum protein analysis and 0.9% of abnormal plasma cells with only t(11; 14) abnormalities upon bone marrow biopsy (Figure 1E). The addition of venetoclax to the treatment regimen of our patient could have been another strategy to delay the aggressive disease course. As compared to MM, PCL patients usually have decreased expression of the adhesion molecules NCAM (neural adhesion molecule/CD56) and LFA-1 (leukocyte function-associated antigen-1), and thus have extramedullary accumulation and progression of PCL. Hence, CD56 expression on tumor cells is used as a prognostic factor marker even in patients who have undergone treatment with bortezomib [6,16]. 

Usually autologous and allogenic stem cell therapy has been associated with longer progression-free survival (PFS) and OS in MM patients but complete remission has been reported in PCL especially of primary form with stem cell therapy although OS was inferior to that of patients with MM [17]. Especially in older patients with PCL, the prognosis of PCL with conventional chemotherapy without novel agents is poor; hence, treatment with novel immunomodulatory drugs and proteasome inhibitors is recommended. Bortezomid rapidly reduces the tumor load, reverses PCL associated complications such as renal failure and hypercalcemia, and has a 69% of overall response rate [18]. Similarly, lenalidomide and pomalidomide with dexamethasone and bortezomid have been used in relapse refractory cases of MM with an overall response rate of 59% with a mean duration of response of 13.6 months [18]. In advanced age patients with or without comorbidities, induction, consolidation and maintenance with bortezomib-based regimen have improved the response rate and OS in sPCL and MM patients [18]. 

Although proteasome inhibitors, immunomodulatory agents and monoclonal antibodies have been able to improve the clinical outcome in MM, clinical remission could still be a challenge with these agents. Our patient failed to achieve remission and was treated with proteasome inhibitors and immunomodulatory agents. Several molecular targets, including surface molecules, adhesion molecules and transmembrane receptors, have been identified for MM. Among these targets, anti-CD38 (isatuximab and daratumab), anti-CD56 (lorvotuzumab mertansine), anti-CD138 (indatuximab) alone or in combination with other immunomodulatory agents and program cell death (PD-1) antibody (nivolumab, pembrolizumab and pidilizumab) in clinical trials showed OS ranging from 39% to 78% with higher response to combination regimen as compared to monotherapy with monoclonal antibody [19,20]. In addition, chimeric antigen receptor T cell (CART) therapy targeting B-cell maturation antigen (BCMA), CD19, KLC and CD138 have shown promising results in phase I clinical trials, especially forrelapse/recurrent MM patients. BCMA-based CART has also shown to eradicate plasmacytoma in some patients [20]. LCAR-B38M CART containing a CAR construct with scFv targeting two BCMA epitopes, VHH1 and VHH2, demonstrated an 88% of overall response rate with minimal adverse events including grade 1 cytokine release syndrome [20].

Our patient had a rapid progression of MM to sPCL and then spread of MM to CNS. To our knowledge, such unique case scenario with unpredictable clinical course of MM has never been reported previously. Being 79-year-old with comorbid conditions, the patient underwent treatment regimen consisting of proteasome inhibitors and immunomodulatory agents but was not able to enroll into clinical trials for treatment with anti-MM antibodies or CART therapy or even bone marrow transplantation. Our case report highlights the clinical scenario where management of MM due to its aggressive clinical course is indeed a great challenge.

## Conclusions

Despite being a rare entity, diagnosis and treatment of sPCL requires careful consideration in cases presenting with recurrent MM, especially in the patients with advanced age and comorbidities. The treatment regimen comprised of combination of novel immunomodulatory agents and monoclonal antibodies has shown improved OS rate. The results from the ongoing clinical trials with CART targeting BCMA, CD19, KLC and CD138 are promising for therapy of refractory/relapsed MM, especially in patients with aggressive clinical course. Our case report underscores the challenge with respect to treatment and management for MM especially when it progresses to sPCL within a short period of time.
